# A Rare Cause of Persistent Blood Loss after Continuous Ambulatory Peritoneal Dialysis Catheter Placement

**DOI:** 10.1155/2020/1309418

**Published:** 2020-02-20

**Authors:** T. Natroshvili, T. Elling, S. A. Dam, M. vd Berg, R. R. H. Nap, R. J. Hissink

**Affiliations:** ^1^Department of Surgery, Treant Health Care Group, Scheper Hospital, Boermarkeweg 60, 7824 AA Emmen, Netherlands; ^2^Department of Internal Medicine, Treant Health Care Group, Scheper Hospital, Boermarkeweg 60, 7824 AA Emmen, Netherlands

## Abstract

The laparoscopic placement of a continuous ambulatory peritoneal dialysis (CAPD) catheter is a widely used method in patients with end stage renal disease (ESRD). The potential complications of this procedure include perforation of intra-abdominal organs, surgical site infection, peritonitis, catheter migration, catheter blockage, port site herniation, and bleeding. In most cases, bleeding is considered to be an early-onset complication because it mostly occurs within the first seven days after surgery. We report a case of a 68-year-old female patient with a previous history of diabetes mellitus, myelodysplastic syndrome, extensive collateral varices, anaemia, and ESRD due to obstructive uropathy caused by retroperitoneal fibrosis, who presented with persistent blood loss after the laparoscopic placement of a CAPD catheter. Duplex ultrasonography showed that the CAPD catheter was transfixing a superficial epigastric varicose vein, a collateral vein, due to the occlusion of the left external iliac vein. Persistent blood loss after inserting a CAPD catheter without previous imaging of abdominal wall vessels is an indication for further diagnostics.

## 1. Introduction

Continuous ambulatory peritoneal dialysis (CAPD) is a widely accepted method of treatment for patients with end stage renal disease (ESRD) [1, 2]. The CAPD catheter can be placed by several techniques, including open surgery, a percutaneous insertion, and a laparoscopic-assisted technique [3, 4]. The potential complications of CAPD catheter placement are migration of the catheter, surgical site infection, peritonitis, obstruction, perforation of intra-abdominal organs, postoperative bleeding, and dialysate leakage. These complications increase health care cost, decrease the quality of life, and potentially contribute to reduced utilization of CAPD [5]. This case report illustrates an unusual complication of a patient developing persistent blood loss after CAPD catheter placement.

## 2. Presentation of Case

A 68-year-old woman with a medical history of diabetes mellitus, myelodysplastic syndrome, extensive collateral varices, anaemia, and ESRD due to obstructive uropathy caused by retroperitoneal fibrosis received a CAPD therapy. The patient had no history of deep venous thrombosis. The CAPD catheter was placed using a laparoscopic technique. The first cuff was placed at the height of the peritoneum in the direction of the Douglas' pouch. The second cuff was placed subcutaneously. No obstructions were found in the catheter when it was flushed with normal saline. The colour of the returning fluid was in accordance with expectations for this procedure. The patient had an uneventful postoperative recovery, except for persistent superficial bleeding at the exit site of the CAPD catheter. The patient was discharged home one day postoperatively. At the time of discharge, the patient had 80 mg acetylsalicylic acid and no other anticoagulant therapy.

Nine weeks after the operation, the exit site remained bleeding superficially, which appeared to be of venous origin due to its slow rate of bleeding and dark red colour. The bleeding persisted intermittently but was not observed at the time of the outpatient visit. Local compression of the wound was given and antiplatelet therapy was stopped.

**Figure 1:**
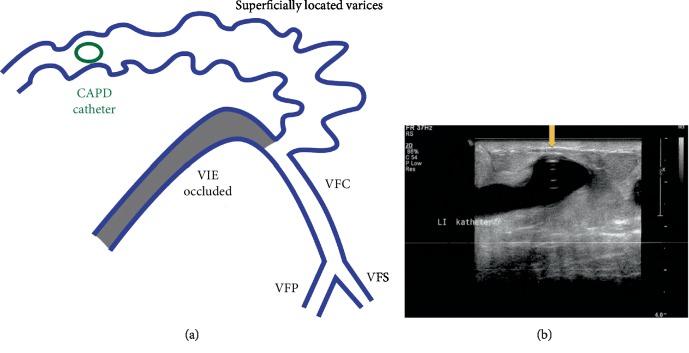
(a) CAPD catheter passing through a large epigastric varicose vein, as visualized with duplex ultrasonography. Schematic presentation of the duplex ultrasonography. The left external iliac vein (VIE) seemed to be completely occluded. The common iliac vein (VIC) cannot be visualized properly. At the height of the mid common femoral vein (VFC), the duplex revealed the existence of various abdominal wall varices, with relatively high flow, which can be followed all the way to the sternum. The CAPD catheter had been located in one of these superficially located abdominal wall varices. VIE: external iliac vein; VIC: common iliac vein; VFC: common femoral vein; VFP: profunda femoris vein; VFS: superficial femoral vein. (b) Duplex ultrasonography showing the position of the CAPD catheter (arrow) in a variceous abdominal wall vein.

However, the bleeding persevered and approximately four months postoperative the patient was presented to the emergency room with increased blood loss and anaemia. Laboratory examination showed decreasing haemoglobin from 5.4 to 4 mmol/L. There were no signs of acute blood loss. Bleeding observed at the exit site of the CAPD catheter was the most plausible cause of the blood loss. Duplex ultrasonography (DUS) of the abdomen was performed, which showed no involvement of the arterial vascular system. However, the catheter appeared to be passing through a large epigastric varicose vein, see Figures 1(a) and 1(b). Furthermore, occlusion in the left external iliac vein was noted. The venous flow was relatively high, as expected in the cases where the superficial vein gains a collateral function. In our case, the vein could be followed up all the way to the sternum. The localisation of the CAPD catheter through the vein caused the persistent blood loss.

A reinsertion of the CAPD catheter was done. The varicose vein was marked preoperatively with a DUS, the incision was made along the catheter, and the vein was exposed. The findings of the DUS were confirmed during the reoperation. It appeared that the varicose vein had been punctured in the subcutaneous trajectory. The catheter was removed and the defects in the vein wall were closed preserving the vein. Another subcutaneous tunnel was created for the catheter. The patient developed mild wound infection during the recovery, which was successfully treated with antibiotics. The patient was discharged two days after the operation. No further bleeding occurred. At six-month follow-up, the patient was well and with no evidence of blood loss due to the CAPD catheter.

## 3. Discussion

One of the known complications of CAPD catheter insertion is postoperative bleeding. Flayou et al. in 2016 described postoperative bleeding as an early-onset complication in 10.5% of the patients, meaning the bleeding started within the first seven days after the operation. Compression was the adequate therapy for these patients. Persistent bleeding was not mentioned as a complication, neither was the existence of superficially located veins [6].

One case report described the eroding of a peritoneal catheter into a mesenteric artery as a cause of hemoperitoneum, which is a result of a bleeding complication that can occur after the placement of a CAPD catheter [7–9]. In several cases, the injury to the inferior epigastric artery has been reported as a major bleeding complication due to CAPD catheter placement [10]. Most of the time, hemoperitoneum and/or postoperative bleeding is noted as a benign complication, with no significant long-term effects on predisposition to peritonitis or patient survival [11]. However, if the bleeding is recurrent, heavy, and/or associated with fever and pain, further diagnostics are required to exclude underlying intra-abdominal pathology.

**Figure 2:**
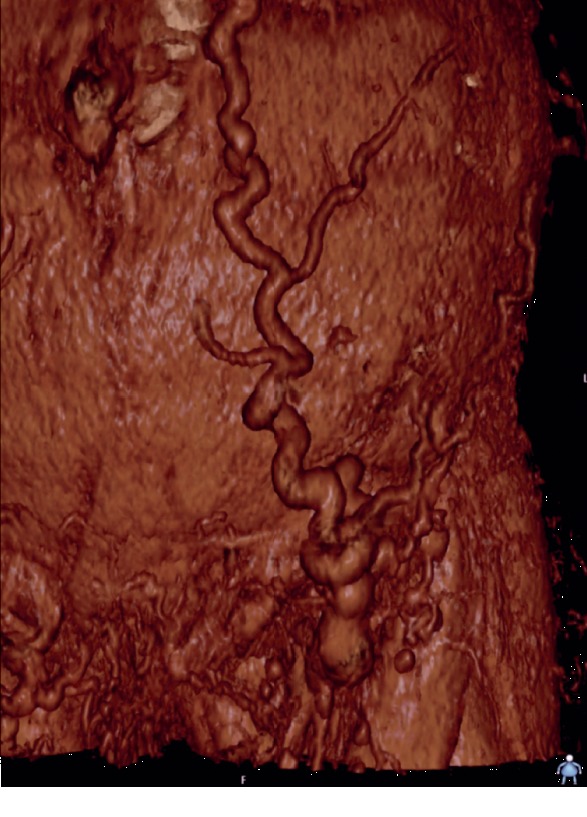
Reconstruction of the abdominal wall with a superficial collateral vein from a CT performed in 2013.

In this exceptional case, the persistent blood loss was due to a superficially located vein which gained the function of a collateral vein because of an asymptomatic iliac vein occlusion which had existed for years. Retrospectively, occlusion and a superficial collateral vein could be seen at a CT angiography performed earlier in 2013, see Figure 2. Due to a compensatory mechanism, this vein developed into a low-pressure, high-flow collateral vein. The CAPD catheter transfixing the vein maintained the bleeding, and obviously there was not enough local pressure increase to tamponade the bleeding.

This case creates an awareness for collateral venous systems in patients with known occlusions of big vessels. Further, it is a good illustration that a further diagnostic investigation is required if blood loss will not decrease and/or will persist for weeks after inserting a CAPD catheter. The early use of diagnostic tools, such as a DUS or a CT with intravenous contrast, in similar cases of persistent blood loss might avoid extra costs or emergency admission and may contribute to a better quality of life.

This case report demonstrates a patient with persistent blood loss due to a CAPD catheter transfixing in a superficially located collateral vein caused by an asymptomatic iliac vein occlusion. We advocate for a careful evaluation of the patient preoperatively, especially in case of a visible abdominal wall varicosity during the physical examination or known occlusion of big vessels which can contribute in the formation of collateral vessels.

Postoperatively, if the blood loss will not decrease and/or will persist for more than one week, we recommend further diagnostic investigation.

It is important to rely on current practical guidelines for patients with ESKD requiring CAPD, and this exceptional case is a good illustration of practical knowledge enrichment [12, 13]. The next generation of nephrologists and surgeons will likely base their decision regarding treatment options by evaluating information obtained through the collection and analysis of big data. Furthermore, the role of virtual surgery to plan and/or evaluate risks related to invasive treatment options will become more and more relevant in complex cases [14, 15].
